# Gas Bacillus Infections

**Published:** 1913-03

**Authors:** 


GAS BACILLUS INFECTIONS.—Walter C. Cramp, New York City (*Annals of Surgery*, October, 1912), reports 187 cases of his own and others. His net conclusions are for sweeping incisions of infected tissues with continuous application of hydrogen peroxide, frequently if not constantly renewed.

